# 4′-[4-(Pyridin-2-yl)phen­yl]-2,2′:6′,2′′-terpyridine

**DOI:** 10.1107/S1600536810039413

**Published:** 2010-10-09

**Authors:** Chao-Yun Zhu

**Affiliations:** a Department of Applied Chemistry, Nanjing College of Chemical Technology, Nanjing 210048, People’s Republic of China

## Abstract

In the title compound, C_26_H_18_N_4_, each ring is almost planar with maximum deviation of 0.012 (5) Å. In the crystal, mol­ecules are stacked by weak C—H⋯π inter­actions, forming a three-dimensional framework.

## Related literature

For the uses and the synthesis of the title compound, see: Arm *et al.* (2006 ▶). For bond-length data, see: Allen *et al.* (1987 ▶).
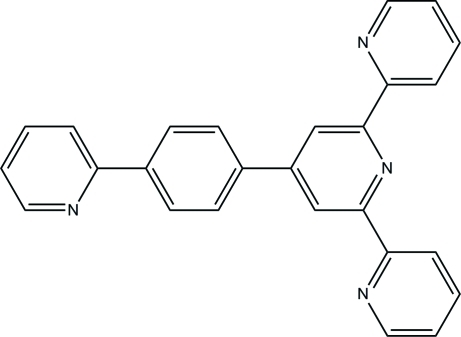


## Experimental

### 

#### Crystal data

C_26_H_18_N_4_
*M* *_r_* = 386.44
Monoclinic, 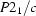

*a* = 11.056 (2) Å
*b* = 16.113 (3) Å
*c* = 11.748 (2) Å
β = 109.05 (3)°
*V* = 1978.2 (7) Å^3^
*Z* = 4
Mo *K*α radiation
μ = 0.08 mm^−1^
*T* = 298 K
0.30 × 0.20 × 0.05 mm

#### Data collection

Enraf–Nonius CAD-4 diffractometer
Absorption correction: ψ scan (North *et al.*, 1968 ▶) *T*_min_ = 0.977, *T*_max_ = 0.996
3778 measured reflections
3585 independent reflections
1576 reflections with *I* > 2σ(*I*)
*R*_int_ = 0.100
3 standard reflections every 200 reflections  intensity decay: 1%

#### Refinement

*R*[*F* ^2^ > 2σ(*F* ^2^)] = 0.069
*wR*(*F* ^2^) = 0.217
*S* = 0.88
3585 reflections
271 parameters
H-atom parameters constrained
Δρ_max_ = 0.16 e Å^−3^
Δρ_min_ = −0.16 e Å^−3^

### 

Data collection: *CAD-4 Software* (Enraf–Nonius, 1985 ▶); cell refinement: *CAD-4 Software*; data reduction: *XCAD4* (Harms & Wocadlo, 1995 ▶); program(s) used to solve structure: *SHELXS97* (Sheldrick, 2008 ▶); program(s) used to refine structure: *SHELXL97* (Sheldrick, 2008 ▶); molecular graphics: *SHELXTL* (Sheldrick, 2008 ▶); software used to prepare material for publication: *SHELXTL*.

## Supplementary Material

Crystal structure: contains datablocks I, Is1. DOI: 10.1107/S1600536810039413/bq2233sup1.cif

Structure factors: contains datablocks I. DOI: 10.1107/S1600536810039413/bq2233Isup2.hkl

Additional supplementary materials:  crystallographic information; 3D view; checkCIF report

## Figures and Tables

**Table 1:** Hydrogen-bond geometry (Å, °) *Cg*1 and *Cg*2 are the centroids of the N2/C12–C16 and N3/C17–C21 rings, respectively.

*D*—H⋯*A*	*D*—H	H⋯*A*	*D*⋯*A*	*D*—H⋯*A*
C7—H7*A*⋯*Cg*1^i^	0.93	2.97	3.675 (5)	134
C25—H25*A*⋯*Cg*2^ii^	0.93	3.19	3.989 (5)	146

Symmetry codes: (i) 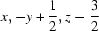; (ii) 
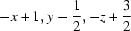
.

**Fig. 1.**
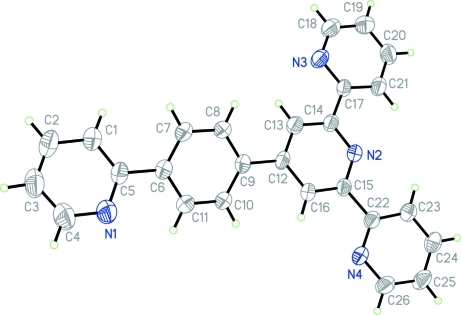
The asymmetric unit of the title compound, with the atom-numbering scheme. Displacement ellipsoids are drawn at the 50% probability level.

**Fig. 2.**
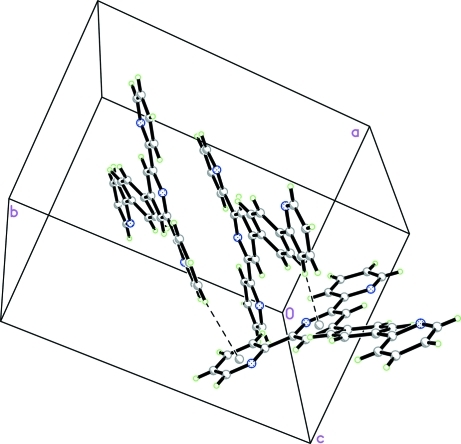
Crystal packing of the title compound.

## supplementary crystallographic information

### Comment

4'-(4-(Pyridin-2-yl)phenyl)-2,2':6',2''-terpyridine, (I), is an important intermediate used to synthesize luminescent transition metal complexes, which are potentially interesting components of new molecular sensors for detection of molecules or ions in aqueous solution (Arm *et al.*, 2006). We report here the crystal structure of the title compound (Fig. 1).

Bond lengths (Allen *et al.*, 1987) and angles of (I) are within normal ranges. In (I), each ring is planar with the max. deviation 0.012 (5) Å. The dihedral angles of the rings A (C1—C5/N1), B(C6—C11), C(C12—C16/N2), D(C17—C21/N3), E(C22—C26/N4) are: A/B = 159.6 (2)°, C/B = 158.5 (2)°, D/A = 4.8 (1)°, E/A = 170.6 (3)°.

In the crystal structure, no obvious hydrogen bond was observed, and molecules were stacked to form a three-dimensional framework by two C—H···π weak interactions, which may be effective for the stabilization of the crystal (Table 1, Fig 2).

### Experimental

The title compound (I) was prepared by the method reported in literature (Arm *et al.*, 2006). Single crystals were obtained by dissolving the title compound (0.5 g, 1.3 mmol) in ethanol (100 ml) and evaporating the solvent slowly at room temperature for about 20 d.

### Refinement

After checking their presence in the difference map, all the H atoms were positioned geometrically [C—H = 0.93 Å] and constrained to ride on their parent atoms, with U_iso_(H) = 1.2U_eq_(C,N).

### Crystal data

**Table:**

C_26_H_18_N_4_	*F*(000) = 808
*M**_r_* = 386.44	*D*_x_ = 1.298 Mg m^−^^3^
Monoclinic, *P*2_1_/*c*	Mo *K*α radiation, λ = 0.71073 Å
Hall symbol: -P 2ybc	Cell parameters from 25 reflections
*a* = 11.056 (2) Å	θ = 9–12°
*b* = 16.113 (3) Å	µ = 0.08 mm^−^^1^
*c* = 11.748 (2) Å	*T* = 298 K
β = 109.05 (3)°	Block, colorless
*V* = 1978.2 (7) Å^3^	0.30 × 0.20 × 0.05 mm
*Z* = 4	

### Data collection

**Table:**

Enraf–Nonius CAD-4 diffractometer	1576 reflections with *I* > 2σ(*I*)
Radiation source: fine-focus sealed tube	*R*_int_ = 0.100
graphite	θ_max_ = 25.3°, θ_min_ = 2.0°
ω/2θ scans	*h* = 0→13
Absorption correction: ψ scan (North *et al.*, 1968)	*k* = 0→19
*T*_min_ = 0.977, *T*_max_ = 0.996	*l* = −14→13
3778 measured reflections	3 standard reflections every 200 reflections
3585 independent reflections	intensity decay: 1%

### Refinement

**Table:**

Refinement on *F*^2^	Primary atom site location: structure-invariant direct methods
Least-squares matrix: full	Secondary atom site location: difference Fourier map
*R*[*F*^2^ > 2σ(*F*^2^)] = 0.069	Hydrogen site location: inferred from neighbouring sites
*wR*(*F*^2^) = 0.217	H-atom parameters constrained
*S* = 0.88	*w* = 1/[σ^2^(*F*_o_^2^) + (0.1*P*)^2^] where *P* = (*F*_o_^2^ + 2*F*_c_^2^)/3
3585 reflections	(Δ/σ)_max_ < 0.001
271 parameters	Δρ_max_ = 0.16 e Å^−^^3^
0 restraints	Δρ_min_ = −0.16 e Å^−^^3^

### Special details

**Table:**

Geometry. All e.s.d.'s (except the e.s.d. in the dihedral angle between two l.s. planes) are estimated using the full covariance matrix. The cell e.s.d.'s are taken into account individually in the estimation of e.s.d.'s in distances, angles and torsion angles; correlations between e.s.d.'s in cell parameters are only used when they are defined by crystal symmetry. An approximate (isotropic) treatment of cell e.s.d.'s is used for estimating e.s.d.'s involving l.s. planes.
Refinement. Refinement of *F*^2^ against ALL reflections. The weighted *R*-factor *wR* and goodness of fit *S* are based on *F*^2^, conventional *R*-factors *R* are based on *F*, with *F* set to zero for negative *F*^2^. The threshold expression of *F*^2^ > σ(*F*^2^) is used only for calculating *R*-factors(gt) *etc*. and is not relevant to the choice of reflections for refinement. *R*-factors based on *F*^2^ are statistically about twice as large as those based on *F*, and *R*- factors based on ALL data will be even larger.

### Fractional atomic coordinates and isotropic or equivalent isotropic displacement parameters (Å^2^)

**Table:**

	*x*	*y*	*z*	*U*_iso_*/*U*_eq_	
N1	0.0183 (4)	0.5691 (3)	−0.2077 (3)	0.0771 (12)	
C1	0.1554 (4)	0.6611 (3)	−0.2631 (4)	0.0634 (12)	
H1B	0.2255	0.6967	−0.2401	0.076*	
N2	0.5599 (3)	0.65373 (19)	0.5784 (3)	0.0471 (8)	
C2	0.0865 (5)	0.6503 (3)	−0.3842 (4)	0.0801 (15)	
H2B	0.1114	0.6769	−0.4433	0.096*	
N3	0.7514 (3)	0.7844 (2)	0.4578 (3)	0.0615 (10)	
C3	−0.0173 (5)	0.6003 (4)	−0.4147 (4)	0.0881 (16)	
H3B	−0.0671	0.5933	−0.4949	0.106*	
N4	0.3280 (3)	0.5058 (2)	0.6038 (3)	0.0554 (9)	
C4	−0.0471 (5)	0.5607 (4)	−0.3254 (4)	0.102 (2)	
H4B	−0.1175	0.5254	−0.3472	0.122*	
C5	0.1198 (4)	0.6189 (3)	−0.1773 (3)	0.0527 (11)	
C6	0.1957 (4)	0.6248 (2)	−0.0466 (3)	0.0482 (10)	
C7	0.2778 (4)	0.6900 (3)	−0.0008 (3)	0.0631 (12)	
H7A	0.2854	0.7321	−0.0521	0.076*	
C8	0.3490 (4)	0.6939 (3)	0.1201 (3)	0.0627 (12)	
H8A	0.4035	0.7388	0.1479	0.075*	
C9	0.3417 (4)	0.6330 (2)	0.2014 (3)	0.0458 (10)	
C10	0.2592 (4)	0.5674 (2)	0.1543 (3)	0.0516 (10)	
H10A	0.2524	0.5247	0.2051	0.062*	
C11	0.1874 (4)	0.5641 (2)	0.0346 (3)	0.0540 (11)	
H11A	0.1316	0.5198	0.0069	0.065*	
C12	0.4183 (4)	0.6392 (2)	0.3317 (3)	0.0451 (10)	
C13	0.5279 (4)	0.6881 (2)	0.3720 (3)	0.0504 (10)	
H13A	0.5561	0.7165	0.3165	0.060*	
C14	0.5960 (4)	0.6950 (2)	0.4949 (3)	0.0464 (10)	
C15	0.4563 (4)	0.6052 (2)	0.5413 (3)	0.0433 (9)	
C16	0.3837 (4)	0.5966 (2)	0.4191 (3)	0.0483 (10)	
H16A	0.3121	0.5623	0.3967	0.058*	
C17	0.7120 (4)	0.7465 (2)	0.5409 (3)	0.0459 (9)	
C18	0.8562 (4)	0.8311 (3)	0.4969 (4)	0.0724 (14)	
H18A	0.8851	0.8572	0.4399	0.087*	
C19	0.9243 (4)	0.8431 (3)	0.6170 (4)	0.0694 (13)	
H19A	0.9967	0.8767	0.6403	0.083*	
C20	0.8825 (4)	0.8045 (3)	0.7007 (4)	0.0650 (12)	
H20A	0.9264	0.8113	0.7824	0.078*	
C21	0.7747 (4)	0.7551 (3)	0.6631 (3)	0.0571 (11)	
H21A	0.7448	0.7283	0.7188	0.069*	
C22	0.4209 (4)	0.5621 (2)	0.6383 (3)	0.0459 (10)	
C23	0.4842 (4)	0.5816 (3)	0.7587 (3)	0.0534 (11)	
H23A	0.5490	0.6212	0.7796	0.064*	
C24	0.4488 (4)	0.5412 (3)	0.8459 (3)	0.0636 (12)	
H24A	0.4879	0.5543	0.9267	0.076*	
C25	0.3555 (4)	0.4814 (3)	0.8129 (3)	0.0638 (12)	
H25A	0.3315	0.4522	0.8704	0.077*	
C26	0.2982 (4)	0.4660 (3)	0.6910 (4)	0.0626 (12)	
H26A	0.2351	0.4254	0.6685	0.075*	

### Atomic displacement parameters (Å^2^)

**Table:**

	*U*^11^	*U*^22^	*U*^33^	*U*^12^	*U*^13^	*U*^23^
N1	0.071 (3)	0.105 (3)	0.053 (2)	−0.025 (2)	0.017 (2)	−0.008 (2)
C1	0.070 (3)	0.073 (3)	0.044 (2)	−0.002 (3)	0.014 (2)	0.000 (2)
N2	0.056 (2)	0.050 (2)	0.0383 (17)	−0.0039 (17)	0.0195 (16)	−0.0027 (15)
C2	0.094 (4)	0.102 (4)	0.044 (3)	0.004 (3)	0.021 (3)	0.009 (3)
N3	0.065 (2)	0.075 (3)	0.048 (2)	−0.011 (2)	0.0235 (19)	0.0061 (18)
C3	0.093 (4)	0.115 (5)	0.047 (3)	−0.009 (4)	0.010 (3)	−0.008 (3)
N4	0.066 (2)	0.065 (2)	0.0406 (19)	−0.010 (2)	0.0247 (17)	0.0004 (17)
C4	0.091 (4)	0.144 (6)	0.062 (3)	−0.045 (4)	0.013 (3)	−0.020 (3)
C5	0.055 (3)	0.065 (3)	0.040 (2)	0.003 (2)	0.018 (2)	−0.003 (2)
C6	0.053 (2)	0.054 (3)	0.041 (2)	0.002 (2)	0.0200 (19)	0.0002 (19)
C7	0.082 (3)	0.068 (3)	0.037 (2)	−0.020 (3)	0.016 (2)	0.006 (2)
C8	0.088 (3)	0.057 (3)	0.042 (2)	−0.024 (3)	0.020 (2)	0.000 (2)
C9	0.050 (2)	0.051 (2)	0.039 (2)	0.003 (2)	0.0194 (19)	−0.0030 (19)
C10	0.061 (3)	0.055 (3)	0.039 (2)	−0.010 (2)	0.017 (2)	0.0022 (19)
C11	0.059 (3)	0.058 (3)	0.050 (2)	−0.011 (2)	0.025 (2)	−0.003 (2)
C12	0.057 (3)	0.049 (2)	0.034 (2)	0.006 (2)	0.0220 (19)	0.0035 (18)
C13	0.057 (3)	0.051 (2)	0.047 (2)	0.001 (2)	0.023 (2)	0.0008 (19)
C14	0.058 (2)	0.046 (2)	0.041 (2)	0.002 (2)	0.0233 (19)	0.0005 (18)
C15	0.051 (2)	0.044 (2)	0.039 (2)	0.003 (2)	0.0202 (19)	−0.0022 (17)
C16	0.058 (3)	0.053 (2)	0.037 (2)	−0.005 (2)	0.0208 (19)	−0.0054 (18)
C17	0.056 (2)	0.047 (2)	0.037 (2)	0.001 (2)	0.0191 (18)	−0.0026 (18)
C18	0.078 (3)	0.084 (4)	0.059 (3)	−0.021 (3)	0.027 (3)	0.003 (3)
C19	0.069 (3)	0.072 (3)	0.071 (3)	−0.014 (3)	0.027 (3)	−0.002 (3)
C20	0.067 (3)	0.074 (3)	0.049 (3)	−0.008 (3)	0.014 (2)	−0.013 (2)
C21	0.063 (3)	0.070 (3)	0.040 (2)	−0.007 (2)	0.020 (2)	−0.003 (2)
C22	0.058 (3)	0.041 (2)	0.044 (2)	0.006 (2)	0.023 (2)	0.0038 (18)
C23	0.062 (3)	0.057 (3)	0.041 (2)	−0.002 (2)	0.017 (2)	0.0040 (19)
C24	0.079 (3)	0.075 (3)	0.033 (2)	−0.001 (3)	0.014 (2)	0.013 (2)
C25	0.076 (3)	0.076 (3)	0.044 (3)	−0.002 (3)	0.025 (2)	0.017 (2)
C26	0.067 (3)	0.072 (3)	0.050 (3)	−0.015 (2)	0.021 (2)	0.008 (2)

### Geometric parameters (Å, °)

**Table:**

N1—C5	1.330 (5)	C10—H10A	0.9300
N1—C4	1.342 (5)	C11—H11A	0.9300
C1—C5	1.376 (5)	C12—C16	1.389 (5)
C1—C2	1.388 (5)	C12—C13	1.392 (5)
C1—H1B	0.9300	C13—C14	1.398 (5)
N2—C15	1.338 (4)	C13—H13A	0.9300
N2—C14	1.348 (4)	C14—C17	1.474 (5)
C2—C3	1.351 (7)	C15—C16	1.404 (5)
C2—H2B	0.9300	C15—C22	1.491 (5)
N3—C18	1.332 (5)	C16—H16A	0.9300
N3—C17	1.339 (4)	C17—C21	1.382 (5)
C3—C4	1.358 (7)	C18—C19	1.379 (5)
C3—H3B	0.9300	C18—H18A	0.9300
N4—C22	1.331 (5)	C19—C20	1.366 (5)
N4—C26	1.339 (4)	C19—H19A	0.9300
C4—H4B	0.9300	C20—C21	1.380 (5)
C5—C6	1.493 (5)	C20—H20A	0.9300
C6—C7	1.378 (5)	C21—H21A	0.9300
C6—C11	1.390 (5)	C22—C23	1.394 (5)
C7—C8	1.382 (5)	C23—C24	1.374 (5)
C7—H7A	0.9300	C23—H23A	0.9300
C8—C9	1.390 (5)	C24—C25	1.372 (6)
C8—H8A	0.9300	C24—H24A	0.9300
C9—C10	1.387 (5)	C25—C26	1.386 (5)
C9—C12	1.492 (5)	C25—H25A	0.9300
C10—C11	1.372 (5)	C26—H26A	0.9300
			
C5—N1—C4	117.5 (4)	C12—C13—H13A	119.6
C5—C1—C2	119.8 (4)	C14—C13—H13A	119.6
C5—C1—H1B	120.1	N2—C14—C13	121.6 (4)
C2—C1—H1B	120.1	N2—C14—C17	116.1 (3)
C15—N2—C14	118.5 (3)	C13—C14—C17	122.3 (3)
C3—C2—C1	118.8 (5)	N2—C15—C16	122.3 (3)
C3—C2—H2B	120.6	N2—C15—C22	115.7 (3)
C1—C2—H2B	120.6	C16—C15—C22	122.0 (4)
C18—N3—C17	117.4 (4)	C12—C16—C15	120.2 (4)
C2—C3—C4	118.4 (5)	C12—C16—H16A	119.9
C2—C3—H3B	120.8	C15—C16—H16A	119.9
C4—C3—H3B	120.8	N3—C17—C21	122.6 (4)
C22—N4—C26	116.9 (3)	N3—C17—C14	116.2 (3)
N1—C4—C3	124.3 (5)	C21—C17—C14	121.3 (3)
N1—C4—H4B	117.9	N3—C18—C19	123.7 (4)
C3—C4—H4B	117.9	N3—C18—H18A	118.2
N1—C5—C1	121.3 (4)	C19—C18—H18A	118.2
N1—C5—C6	117.1 (4)	C20—C19—C18	118.2 (4)
C1—C5—C6	121.5 (4)	C20—C19—H19A	120.9
C7—C6—C11	116.8 (4)	C18—C19—H19A	120.9
C7—C6—C5	121.9 (4)	C19—C20—C21	119.5 (4)
C11—C6—C5	121.4 (4)	C19—C20—H20A	120.3
C6—C7—C8	121.2 (4)	C21—C20—H20A	120.3
C6—C7—H7A	119.4	C20—C21—C17	118.6 (4)
C8—C7—H7A	119.4	C20—C21—H21A	120.7
C7—C8—C9	122.2 (4)	C17—C21—H21A	120.7
C7—C8—H8A	118.9	N4—C22—C23	123.0 (3)
C9—C8—H8A	118.9	N4—C22—C15	116.9 (3)
C10—C9—C8	116.2 (3)	C23—C22—C15	120.1 (4)
C10—C9—C12	122.6 (3)	C24—C23—C22	118.6 (4)
C8—C9—C12	121.2 (4)	C24—C23—H23A	120.7
C11—C10—C9	121.6 (4)	C22—C23—H23A	120.7
C11—C10—H10A	119.2	C25—C24—C23	119.6 (4)
C9—C10—H10A	119.2	C25—C24—H24A	120.2
C10—C11—C6	122.0 (4)	C23—C24—H24A	120.2
C10—C11—H11A	119.0	C24—C25—C26	117.7 (4)
C6—C11—H11A	119.0	C24—C25—H25A	121.1
C16—C12—C13	116.6 (3)	C26—C25—H25A	121.1
C16—C12—C9	121.7 (4)	N4—C26—C25	124.2 (4)
C13—C12—C9	121.7 (3)	N4—C26—H26A	117.9
C12—C13—C14	120.8 (3)	C25—C26—H26A	117.9
			
C5—C1—C2—C3	2.2 (7)	C12—C13—C14—C17	−179.8 (3)
C1—C2—C3—C4	−2.1 (8)	C14—N2—C15—C16	−0.6 (5)
C5—N1—C4—C3	−0.8 (8)	C14—N2—C15—C22	−179.3 (3)
C2—C3—C4—N1	1.5 (10)	C13—C12—C16—C15	1.2 (5)
C4—N1—C5—C1	0.8 (7)	C9—C12—C16—C15	−178.6 (3)
C4—N1—C5—C6	−177.2 (4)	N2—C15—C16—C12	0.0 (6)
C2—C1—C5—N1	−1.6 (7)	C22—C15—C16—C12	178.6 (3)
C2—C1—C5—C6	176.3 (4)	C18—N3—C17—C21	0.8 (6)
N1—C5—C6—C7	−161.0 (4)	C18—N3—C17—C14	−179.9 (4)
C1—C5—C6—C7	21.0 (6)	N2—C14—C17—N3	176.9 (3)
N1—C5—C6—C11	19.6 (6)	C13—C14—C17—N3	−2.1 (5)
C1—C5—C6—C11	−158.4 (4)	N2—C14—C17—C21	−3.7 (5)
C11—C6—C7—C8	0.5 (6)	C13—C14—C17—C21	177.2 (4)
C5—C6—C7—C8	−178.9 (4)	C17—N3—C18—C19	−0.8 (7)
C6—C7—C8—C9	−0.1 (7)	N3—C18—C19—C20	0.5 (7)
C7—C8—C9—C10	0.4 (6)	C18—C19—C20—C21	0.0 (7)
C7—C8—C9—C12	−179.2 (4)	C19—C20—C21—C17	0.0 (6)
C8—C9—C10—C11	−1.1 (6)	N3—C17—C21—C20	−0.4 (6)
C12—C9—C10—C11	178.4 (4)	C14—C17—C21—C20	−179.6 (4)
C9—C10—C11—C6	1.7 (6)	C26—N4—C22—C23	−1.4 (6)
C7—C6—C11—C10	−1.3 (6)	C26—N4—C22—C15	178.7 (3)
C5—C6—C11—C10	178.1 (4)	N2—C15—C22—N4	−172.0 (3)
C10—C9—C12—C16	−21.2 (6)	C16—C15—C22—N4	9.3 (5)
C8—C9—C12—C16	158.4 (4)	N2—C15—C22—C23	8.0 (5)
C10—C9—C12—C13	159.0 (4)	C16—C15—C22—C23	−170.6 (3)
C8—C9—C12—C13	−21.4 (6)	N4—C22—C23—C24	−0.3 (6)
C16—C12—C13—C14	−1.8 (5)	C15—C22—C23—C24	179.6 (4)
C9—C12—C13—C14	178.0 (3)	C22—C23—C24—C25	1.9 (6)
C15—N2—C14—C13	0.0 (5)	C23—C24—C25—C26	−1.6 (6)
C15—N2—C14—C17	−179.0 (3)	C22—N4—C26—C25	1.7 (6)
C12—C13—C14—N2	1.3 (6)	C24—C25—C26—N4	−0.2 (7)

### Hydrogen-bond geometry (Å, °)

**Table:**

Cg1 and Cg2 are the centroids of the N2/C12–C16 and N3/C17–C21 rings, respectively.

**Table:**

*D*—H···*A*	*D*—H	H···*A*	*D*···*A*	*D*—H···*A*
C7—H7A···Cg1^i^	0.93	2.97	3.675 (5)	134
C25—H25A···Cg2^ii^	0.93	3.19	3.989 (5)	146

Symmetry codes: (i) *x*, −*y*+1/2, *z*−3/2; (ii) −*x*+1, *y*−1/2, −*z*+3/2.
